# The clinical presentation and early outcomes of necrotizing fasciitis in a Ugandan Tertiary Hospital- a prospective study

**DOI:** 10.1186/1756-0500-7-476

**Published:** 2014-07-28

**Authors:** John Magala, Patson Makobore, Timothy Makumbi, Sam Kaggwa, Edris Kalanzi, Moses Galukande

**Affiliations:** 1 Department of Surgery, Mulago National Referral Hospital, Kampala, Uganda; 2 Department of Surgery, Makerere University College of Health Sciences, Kampala, Uganda

**Keywords:** Necrotizing fasciitis, Clinical presentation and early outcome

## Abstract

**Background:**

Necrotizing fasciitis is an infectious process characterized by rapidly progressing necrosis of superficial fascia and subcutaneous tissue with subsequent necrosis of overlying skin.

Necrotizing fasciitis is a rare but fatal infection. The worldwide incidence is at 0.4 per 100,000. Mortality is up to 80% with no intervention, and 30-50% with intervention. Delay in intervention is associated with poor outcome. The risk factors for necrotizing fasciitis are diabetes mellitus, HIV, malignancy, illicit drug use, malnutrition among others.

The aim of this study was to describe the clinical presentation and early outcomes of necrotizing fasciitis amongst Ugandan patients.

**Methods:**

A prospective descriptive case series study conducted at Mulago National Referral and Teaching hospital from 5^th^ January to 30^th^ April 2011. Patients with necrotizing fasciitis were consecutively recruited after clinical evaluation, laboratory and microbiological tests were performed. Aggressive debridement was done and broad-spectrum antibiotics administered. Patients were followed up on surgical wards. Ethical approval was obtained.

**Results:**

Thirty five patients were recruited over a 4 months period. More males were affected with, M: F 3:1. The 20-40 years age group was most affected. Attainment of healthy granulation tissue took 19 days on average. Mortality rate was 14% (5/35). Limbs were the most affected body parts 20/35 (57%), the scrotum and perineum (23%). Among infants the scalp was the most affected. Co-morbidities included HIV 8/35 (17%), and DM (5%) among others. The commonest organisms were gram negative. Split skin grafting was necessary in 74% (26/35) of patients.

**Conclusion:**

There were a high number of patients with necrotizing fasciitis; it was associated with low mortality but high morbidity (long hospital stay). There was a high preponderance to males and limbs were the more affected body parts.

## Background

Necrotizing fasciitis (NF) is a life threatening insidiously advancing or sometimes fulminant soft tissue infection characterized by widespread fascial necrosis. Bisno et al. defined it as a deep seated infection of the subcutaneous tissue and superficial fascia that results in progressive destruction of the same [1]. Delayed intervention results in extremely high mortality rate (~80 to 100%), and even with intervention mortality rates remain approximately 30 to 50% [2]. It has been for centuries and some of the worst outbreaks have been seen in war-torn areas and military hospitals. Necrotizing fasciitis was first described in 1952 by Wilson [3].

Morbidity is also high in spite of the aggressive treatment. Diagnosing necrotizing fasciitis can be difficult in the early stages due to the paucity of specific cutaneous signs and this can delay treatment [4,5]. It must be diagnosed early and treated consistently and aggressively for better outcomes [4].

Necrotizing fasciitis is not a reportable illness. It affects any age group and has no sex predilection [6]. It affects any part of the body [5]. A clear trigger can be identified in more than 75% cases [7]. Necrotizing fasciitis on the whole is higher in populations with low socio-economic status in Africa and relatively uncommon in Europe, Canada and USA. It is often in advanced stage at presentation [8,9].

With the increase in immunosuppressive conditions (such as diabetes mellitus, cancer, alcoholism, organ transplantation, and HIV), necrotizing fasciitis has been seen to increase [10].

Local trauma to the affected site has been found commonly as a portal of entry for bacteria that initiate the infectious process [11].

The International Classification of Diseases (ICD), tenth revision [12] makes no distinction in NF types however, other sources have classified necrotizing fasciitis into types I, II, and III. Type I is caused by synergistic aerobic and anaerobic bacteria found at culture of necrotic tissue and typically affects the perineum and trunk mostly in immune suppressed individuals, intravenous drug users, diabetics, and as a post-operative complication; and Type II by Group A beta-hemolytic streptococcus and typically occurs in limbs in healthy people with no comorbidities [13,14]. Type III is caused by Clostridia, and saltwater organisms like Vibrio vulnificus or Aeromonas hydrophilia. Necrotizing fasciitis involving the perineum, and genitalia (a type 1 variant) is described as Fournier’s gangrene after the French venereologist, Jean Alfred Fournier [10].

The exact worldwide incidence of necrotizing fasciitis is not known. Poromanski reported an incidence of 0.4 cases per 100,000 [15]. Prevalence is reported higher in the extremes of age. Most studies carried out have looked at few patients spread over several years [16,17].

Poor economic conditions, delays in referral, long distance to tertiary hospital were found to be the main problems patients had to face [5].

Patients with necrotizing fasciitis need admission for some time for complete recovery since they require aggressive debridement, intravenous antibiotics, wound care, and wound coverage. Prompt recognition, urgent radical surgical debridement and use of appropriate antibiotics are the mainstays of management. Despite antibiotics and debridement, mortality rate of Necrotizing fasciitis remains high [10,11]. In Uganda, no study on necrotizing fasciitis has been published.

The purpose of this study was to describe presentation, and early outcomes of NF treatment among patients.

## Methods

### Study design

This was a prospective descriptive case series study

### Study setting

The study was carried out at Mulago Hospital, Kampala, Uganda, the national referral and Makerere University teaching hospital, from 5^th^ January to 30^th^ April 2011.

### Study population

All patients with necrotizing fasciitis who attended the emergency and surgical wards, or were transferred from other wards in the hospital during the study period

Patients of all ages and gender, presenting with signs and symptoms suggestive of necrotizing fasciitis in the emergency surgical ward and admitted on the general surgical wards during the study period were included.

Patients with NF who gave written informed consent prior to recruitment into the study. For patients below the age of 18 years and above 12 years a child assent was sought after the attending adult had given written consent.

Patients with NF who requested for discharge before samples were taken were excluded.

### Recruitment procedure

Patients who fulfilled the inclusion criteria were enrolled consecutively. A careful and detailed history was taken and physical examination done. The history included demographic details, the details of the pain and its degree, the site of the disease, duration of the disease, events prior to onset of the disease like skin infections in adjacent sites, trauma (surgical or other) to the affected area. Other information obtained were: past medical history of diabetes mellitus, HIV infection, and malignancy, surgical history of organ transplantation or other surgery, history of long-standing alcohol use (chronic continual drinking or periodic consumption of alcohol which is characterized by frequent episodes of intoxication in a year) and chronic steroid or illicit drug use was sought.

Physical examination was done starting with the general, then systemic examination: noting respiratory rate, heart rate, central nervous system (level of consciousness), and musculoskeletal system.

Examination of the affected site was done noting the site and extent of the disease and looking out for signs and symptoms suggestive of NF. Clinical diagnosis was determined by the criteria designed by Chin-Ho et al. [15].

The following clinical features of necrotizing fasciitis were elicited: extreme tenderness to palpation in early stages (extending beyond the apparent area of skin involvement), erythema, swelling, and warm to palpation. These can subdivided by stage, in stage 1; blister or bullae formation (serous fluid), skin fluctuance and skin induration occurs. In stage 2 it progresses to hemorrhagic bullae, skin anesthesia, crepitus and skin necrosis with dusky discoloration before progressing to frank gangrene in stage 3.

Typical while debriding there is a presence of gray necrotic fascia with lack of resistance to blunt dissection and a clear absence of bleeding during surgical dissection. In addition there is a presence of foul smelling ‘dishwater’ pus at surgery. Fever, hypotension, tachycardia, semiconscious/unconscious are often accompanying signs in systemic evaluation.

Patients confirmed with a diagnosis of NF from clinical findings and laboratory findings underwent resuscitation, aggressive surgical debridement of all evidence of gangrene till viable tissue was seen under general anaesthesia. Intravenous broad spectrum antibiotics (ceftriaxone 4 g/day and metronidazole 1.5 g/day or in doses weight-adjusted for the young patients) were instituted as culture and sensitivity results were awaited.

Following the first debridement the affected site for patients with a final diagnosis of NF was revisited 24 hours later to assess the need for a repeat debridement. Alternate day cleaning of the wounds with saline and dressing was done until good granulation tissue was seen to have been formed. Follow up for early outcomes was at three weeks from onset of illness and entailed noting the deaths due to NF [16] and the cause of death was noted, survival was noted at three weeks from onset of illness. The duration between debridement and attainment of healthy granulation tissue and the need for wound coverage were also noted.

### Specimen collection

Blood (5mls) for the following investigations was drawn from each participant (put in a sequestrine bottle): CBC, creatinine, serum albumin, random blood glucose, and HIV serology.

Tissue biopsies were taken off at the margin of the apparently normal skin and the affected site by making a deep incision (2 cm) up to and including the deep fascia. Noting the ease with which this layer separates from the underlying layers and the lack of bleeding at the incision site.

Aseptic technique was observed. Tissue biopsy specimens were transported in sterile containers with formalin to the histopathology laboratory for hematoxylin-eosin staining. Pus was taken off, this was aspirated using a sterile syringe that was then tightly capped to prevent air entry and transported to the microbiology laboratory immediately in both aerobic and anaerobic conditions for gram staining, and culture and sensitivity tests.

Tissue samples for culture were placed in degassed bags and sealed. Specimens were plated as soon as they arrived in the laboratory. Cultures (anaerobic) were placed in an environment that was free of oxygen, at 35°C for at least 48 hours before the plates were examined. Organisms were identified by their colonial and microscopic morphology.

### Ethical considerations

Ethical approval was obtained from Makerere School of Medicine Research and Ethics Committee (SoMREC).

All participants provided written informed consent.

## Results

The study was conducted, from 5^th^ January to 30^th^ April 2011. Thirty five patients were recruited. Of these, 26(74%) were male, and 9 (26%) female. The female: male ratio was 1:3 as shown in Table 1.

**Table 1:** Baseline characteristics of NF patients, a Ugandan study

	**Characteristic**	**Number (%)**
**Gender**	Female	9 (26)
	Male	26 (74)
**Age**		
Paediatric	<1 month	3
	<1	1
	1-5 years	0
	5-18 years	3
	> 18 - 40 years	15 (43)
Adult	≥ 41 years	12 (34)
Median (IQR) years^†^	32(25)	
Range years	1 week to 89 years	
**Duration of symptoms(days)**	1-7	17 (49)
	>7	18 (51)
**Inciting event**	Adjacent infection	23 (66)
	Trauma	11 (31)
	Surgery	1 (3)
**Survival**	Died	14 (40)
	Survived	21 (60)
**Body part affected**	Median surface area affected (IQR)	N = 35 (%)
Head and neck	7 (0.4-7)	3 (9%)
Upper limbs	5 (4-9)	3 (9%)
Trunk	2 (0.8-3.5)	4 (11%)
Scrotum and perineum	0.7 (0.4-1)	8 (23%)
Lower limbs	2.3 (0.8-16)	17 (49%)

^†^Median age of the study population.

### Factors associated with necrotizing fasciitis

**Figure 1:**
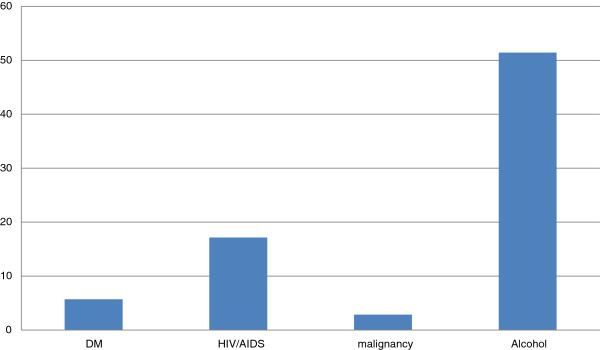
Risk factors of necrotizing fasciitis.

The risk factors of NF included alcohol intake (51%), HIV/AIDS (17%), DM (6%) and, malignancy (3%) as shown in Figure 1.

### Clinical features

Patients in this study complained of the following painful swelling, skin colour changes, blister or bullae formation, foul discharge, and fever. One patient reported no history of skin necrosis and ulceration at presentation.

Physical findings included: site swelling, tenderness, and foul discharge. The body parts affected were: head and neck, upper limbs, trunk, scrotum and perineum and lower limbs as shown in Table 1. Twenty four study subjects (69%) presented with pyrexia of more than 37.5°C in the axilla, and 42% had a tachycardia of > 90beats/minute. Only 6% had low systolic blood pressure of <80 mmHg and 34% had crepitus. Thirty four (97%) haemodynamically stable patients underwent debridement of the necrotic tissue. The operative findings included grayish necrotic fascia, lack of resistance to dissection, and lack of bleeding of fascia. Repeat debridement was done once for three patients (8%) on follow up on the ward.

**Figure 2:**
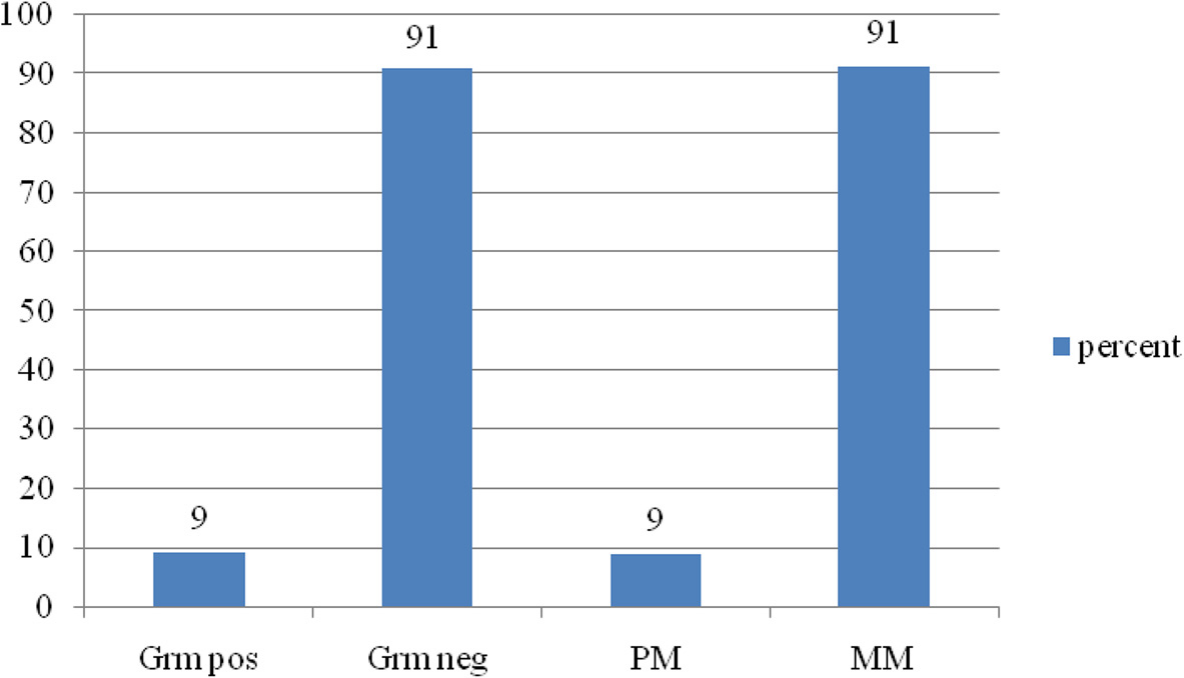
Microbiologic findings. Grm pos=gram positive, Grm neg= gram negative, PM=polymicrobial, MM=monomicrobial.

As indicated in Figure 2, gram positive organisms included enterococcus faecalis, corynebacteria species, and staphylococcus aureus. Gram negative organisms included eschericia coli (E.coli), klebsiella pneumonia, proteus mirabilis, providentia stuarti, pseudomonas aeruginosa, Mycobacterium species, providentia rettgeri, klebsiella oxotyica, and acinetoobacter baumanii.

Some organisms were resistant to commonly used and available antibiotics in this setting like third-generation cephalosporins but sensitive to imipenem amd amikacin.

### Diagnosis

The diagnosis of necrotizing fasciitis was made basing on history, clinical features and investigative findings. Two patients (6%) with infection involving the perineum had perianal sepsis in addition.

### Outcomes of necrotizing fasciitis

There was generally a good outcome of patients with necrotizing fasciitis (86%). There were 5 deaths (14%), four males and one female. The dead were 23 to 48 years old (mean 35 years). Three of these patients were HIV positive, one was a diabetic and these presented to hospital on average after 11 days of onset of the infection and died on average 15 days after onset of illness, and one had a fulminant course of the illness and died within three days of the diagnosis of necrotizing fasciitis following a herniorrhaphy. The cause of death was septicemic shock in these patients.

The duration of attainment of healthy granulation in days among survivors was on average 19 (sd 7, mean = 20, interquartile range =7). The patients who died had not attained healthy granulation tissue at time of death.

### Wound care

The need for wound coverage after attainment of healthy granulation tissue was necessary for 26 patients (74%). The 8 patients who did not require wound closure or coverage had a surface area of wound of less than 1% using the Lund-Browder chart [18]. One patient underwent an amputation of the lower half of the leg which was found to be life-threatening. Patients with a surface area of the wound of more than 1% required wound closure or coverage.

## Discussion

We set out to describe NF as it presents at a sub Saharan African tertiary hospital. A large number of NF was seen relative to the study duration. Previous studies done elsewhere had more men with NF than females [10,19], though some others showed no gender predilection, the actual incidence remains unknown. Neonates and the elderly alike were affected by the disease in this study as was in other studies [6,10,20]. The extremes of ages are probably affected by virtue of their comparably low immunity.

### Factors associated with necrotizing fasciitis

The commonest inciting event was infection representing 66% of all cases (pimples, pustules, paronychia, vesicles, perianal abscess, and nodules). An inciting event as the cause of NF is cites in various studies [19,21]. These infections are common occurrences but their ultimate management in the face of other risk factors may explain the progress to necrotizing fasciitis. Neonates especially are predisposed to skin infections which may progress to a fulminant NF.

The other inciting event in this study was trauma (31%) which included road traffic crashes, trauma by nails, blunt implements, rat bite, pressure sores from Plaster of Paris cast, and surgery (3%). Various studies reported trauma among the inciting events [10,12,22]. In Rea’s series [23] trauma was the leading inciting event.

Other, predisposing risk factors included alcohol abuse (51%), HIV/AIDS (18%), DM (6%), malignancy (3%). Earlier studies found the same risk factors common [6,18,24]. Other risk factors mentioned included vascular disease, obesity, and old age. The HIV prevalence in the Ugandan general population is 7% [25].

Delayed diagnosis and intervention leads to extensive tissue destruction which leads to prolonged hospital stay and increased mortality [16]. In this study, the average hospital stay was 10 days.

### Clinical features

Limbs were mainly affected (60%) among adults in this study followed by the perineum, trunk, head and neck as was in other studies [5,6,10,12].

Most of patients presented in the later stages of illness. This was attributed to delay in referral, poor social economic condition, long distance to tertiary hospital as in other studies [5,15,19].

The pathognomonic sign present in all patients in this study was superficial fascial necrosis which on blunt dissection sheared away without resistance and hardly bled. Thompson et al. described similar features.

Neonates scalps had the largest surface area affected, followed by limbs, trunk and the scrotum and perineal region. Different body parts on the whole had widely differing surface areas of involvement. The surface area affected ultimately will affect morbidity, hospital stay, the need for wound closure or coverage, and survival [5,19]. The patient with the surface area of >16% succumbed to the illness. Wounds with surface area of less than 1% did not require wound coverage.

Among young adults and the middle-aged males the lower limbs and perineo-scrotal region were the most commonly affected body parts. Among the elderly patients the lower limbs were commonly affected.

### Microbiologic findings

Contrary to most other studies, in our study aetiologic agents were mostly monomicrobial. Both gram positive and gram negative micro-organisms were identified; 9% cultures of pus yielded polymicrobial growths and 91% monomicrobial. The most common organisms were E.coli as is in other studies [6,19]. Some organisms’ isolates were resistant to commonly used and available antibiotics for use in this environment like ciprofloxacin, ceftriaxone, and gentamicin. Some cultured organisms such as E.coli, and klebsiella pneumonia were found to have extended spectrum beta-lactamase activities and were only sensitive to antibiotics such as imipenem, amikacin which are quite expensive. Use of antibiotics prior to admission may explain the sensitivity patterns observed in this study. Prior use may perhaps contribute to the monomicrobial picture.

### Management and outcomes of necrotizing fasciitis

NF mortality was 14%, similar to other studies [15,19,24]. The cause of death was septic shock in all the five patients. Advanced systemic toxicity results in septicemic shock with its sequelae of renal shutdown, acute respiratory distress syndrome (ARDS), disseminated intravascular coagulopathy (DIC), severe irreversible hypotension, toxic effects on the myocardium and ultimate death. Death is usually immediately attributed to overwhelming sepsis, diabetic complications, and hemodynamic collapse [6].

All five deaths had E.coli infections. Three had extended spectrum beta-lactamase activity.

Other contributing factors could have been HIV/AIDS, and DM. On average these patients (who died) presented 10 days after onset of symptoms. Necrotizing fasciitis can, in some cases, be fulminant and lead to death in a short period of time, 18 hours according to Amanda Hu [26] or within 24 to 48 hours especially if the patient arrives late in the course of the disease according to Cree et al. [10].

Repeated debridement were done once for three patients until all necrotic tissue was done away with Damiano et al. recommend this [24].

The duration between debridement and attainment of healthy granulation tissue was due to poor healing ability in patients with co-morbidities, poor nutrition, and extensive infection. In a study by Thompson et al. [6] patients attained healthy granulation tissue on 25^th^ day after debridement and were discharged to return for split skin grafting at a later date.

In this study 74% of the patients required wound closure. This would include simple closure, partial thickness skin grafting. The need for wound coverage depended on the wound surface area. Wounds with a percent surface area of 1% and over needed wound coverage. Those with less than 1% surface area wounds healed by secondary intention [5].

One patient underwent amputation due to gangrene involving the foot. This was because the limb had become a threat to life. Amputation is recommended if the limb infection is life threatening [19,24].

Accurate assessment and timely interventions are critical in treatment of patients affected with NF for a favourable outcome.

### Study limitations

Since no MSRA was cultured, we contend that the intra hospital transfers were not hospital acquired infections.

In a few cases, sterile cotton swabs were used to collect anaerobic samples, this is known to be detrimental to some anaerobes (cotton fibres). It was not possible to conclusively ascertain prior use of antibiotics. This may have contributed to the mono-microbial picture. Even though the number of NF patients was high for such a short period of time, the number was small to make some inferences and sub analyses like for HIV.

## Conclusion

NF numbers were high and NF was associated with low mortality and high morbidity (long hospital stay). There was preponderance to males, limbs and the perineum. Aetiologic agents were mostly monomicrobial.

## Competing interests

The authors declare that they have no competing interests.

## Authors’ contributions

SK and EK developed the concept, JP collected and analysed data. PM and MG prepared the Manuscript. All authors read and approved the final manuscript.
